# Efficacy and Safety of Penetrating Keratoplasty and Deep Anterior Lamellar Keratoplasty in Corneal Macular Dystrophy: A Systematic Review and Meta-Analysis

**DOI:** 10.1155/joph/8867750

**Published:** 2025-08-06

**Authors:** Abdelaziz A. Awad, Abdelrahman M. Elettreby, Ahmed A. Abo Elnaga, Mohamed A. Alsaied, Dalia Kamal Ewis, Yousef R. Alnomani, Fatma Mohammed, Mazen M. Sinjab, Abdulla Turki Alsubaey, Zaki Shannak, Hashem Abu Serhan

**Affiliations:** ^1^Faculty of Medicine, Azhar University, Cairo, Egypt; ^2^Faculty of Medicine, Mansoura University, Mansoura, Egypt; ^3^Faculty of Medicine, Beni Suef University, Beni Suef, Egypt; ^4^Faculty of Medicine, Benha University, Benha, Egypt; ^5^Faculty of Medicine, Aswan University, Aswan, Egypt; ^6^Department of Ophthalmology, Dr. Sulaiman Al Habib Hospital, DHCC, Dubai, UAE; ^7^Sinjab Academic Consultancy FZE, Dubai, UAE; ^8^International Keratoconus Society, Muscat, Oman; ^9^Department of Ophthalmology, Hamad Medical Corporation, Doha, Qatar

**Keywords:** corneal macular dystrophy, corneal transplant, deep anterior lamellar keratoplasty, penetrating keratoplasty, stromal dystrophy

## Abstract

**Purpose:** To compare efficacy and safety for deep anterior lamellar keratoplasty (DALK) versus penetrating keratoplasty (PK) for macular corneal dystrophy.

**Methods:** Following PRISMA guidelines, we searched four electronic databases (PubMed, Scopus, Cochrane Library, and Web of Science) to identify eligible studies reported up to January 2024. Using STATA 17, we reported outcomes as log risk ratios (log RRs) or mean difference (SMD) and confidence intervals (CIs). A *p* value ≤ 0.05 is considered statistically significant.

**Results:** DALK was superior to PK in terms of BCVA (Hedge's g: −0.32 with 95% CI [−0.64, −0.01], *p*=0.05), PK was associated with a higher risk of graft rejection in comparison with DALK (log RR: 1.21 with 95% CI [0.25, 2.18], *p*=0.01), and there was no difference between PK and DALK in terms of risk of glaucoma, cataract (log RR: −0.02 with 95% CI [−1.00, 0.95], *p*=0.96), and (log RR: 0.09 with 95% CI [−0.53, 0.71], *p*=0.78). The pooled data were homogeneous (*I*^2^ = 0%; *p*=0.84), respectively.

**Conclusion:** This study suggests that PK, compared to DALK, is associated with worse visual outcomes, with a lower risk of recurrence and a higher risk of graft rejection in macular corneal dystrophy patients.

## 1. Introduction

Macular corneal dystrophy (MCD) is an autosomal recessive disorder affecting both eyes and may result in significant discomfort in vision. Patients with MCD present with multiple greyish-white stromal opacities that have hazy and indistinct borders, extending from the limbus to the other. Functional visual acuity can be lost as the corneal opacity density slowly increases, involving the visual axis. Therefore, performing keratoplasty is necessary to restore the vision and transparency of the cornea [1].

Penetrating keratoplasty (PK) has been considered the definitive choice for the treatment of different corneal pathologic features, including the dystrophies of the corneal stroma [1, 2]. For corneal diseases that do not affect the endothelium, such as stromal scars and dystrophies, deep anterior lamellar keratoplasty (DALK) is the first option for their treatment [3–5]. The DALK is associated with a decreased risk of rejection of the endothelial graft preserving the density of the endothelial cells [6–9]. This may be due to the involvement of the deeper layers of the cornea, making it a preferred option by the surgeons. However, some surgeons believe in the unsuitability of DALK for MCD treatment, explaining that the involvement of the endothelium and stroma as well as the Descemet membrane's fragility in MCD would lead to higher rates of endothelial cell attrition after DALK and interface opacities [4, 10].

No meta-analyses compared PK to DALK for MCD, so we aimed to compare both surgeries in terms of efficacy and safety in the management of MCD.

## 2. Materials and Methods

**Figure 1:**
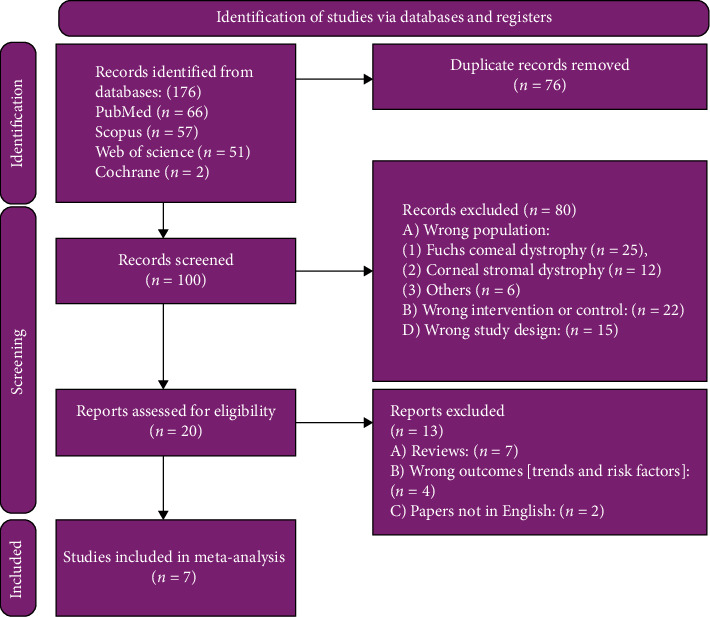
PRISMA flow diagram.

We conducted a systematic review and meta-analysis according to the Preferred Reporting Items for Systematic Reviews and Meta-analysis (PRISMA) strategy and Cochrane's Handbook of Systematic Reviews and Intervention [11, 12] (Figure 1). Our protocol was registered on PROSPERO (CRD42024558024). The need for institutional review board (IRB) approval was exempted due to the nature of our study.

### 2.1. Inclusion Criteria

We formulated the search query according to the PICO framework [13]: The population included patients diagnosed with MCD; the intervention was PK; the comparison was DALK, and the main outcome was best corrected visual acuity (BCVA), endothelial cell density (cells/mm^2^), graft rejection, and recurrence. Secondary outcomes included postoperative complications. No restrictions were made based on the date of publication of the paper.

Articles were included if they were all randomized controlled trials and observational studies comparing PK to DALK while they were excluded if they had one of the following criteria (1) non-English written papers, (2) case reports, (3) series, (4) theses, (5) reviews, (6) in vitro studies, (7) animal studies, (8) conference abstracts, (9) editorials, (10) and studies of unpublished data.

### 2.2. Search Strategy

On 9th January 2024, we searched the following databases: PubMed, Scopus, Web of Science (WOS), and Cochrane. The search strategy is available in the Supporting Appendix.

Two authors (A.A.A. and Y.R.A.) did the title and abstract screening and removed the duplicates and ineligible studies using the Rayyan website [14], then they conducted a full-text screening manually using an Excel sheet to include the studies that met our eligibility criteria. Disagreements amongst reviewers were resolved through extensive discussion, and in cases where a resolution could not be reached, the senior author (H.A.S.) was consulted.

### 2.3. Data Extraction and Management

Using a standardized data extraction sheet two authors (A.M.E. and M.A.A.) extracted the data from the included studies; 3 main sections were made: (1) The baseline sheet included study ID, number of patients who underwent each type of keratoplasty, age in years, gender, primary diagnosis, and postoperative follow-up duration. (2) The summary sheet included study ID, study design, country, number and name of centers, total participants and eyes, duration of receiving standard therapy, main inclusion criteria, primary outcome, and follow-up duration. (3) The outcome sheet included study ID, BCVA, endothelial cell density (cells/mm^2^), graft rejection, recurrence, and postoperative complications.

### 2.4. Risk of Bias Assessment

To assess the quality of the included studies, two authors (A.M.E. and F.M.) independently used the Newcastle Ottawa Scale (NOS) [15]. Only one randomized controlled trial was assessed using ROB-2 [16] (Supporting Tables 1S and 2S).

### 2.5. Statistical Analysis

We conducted a meta-analysis to quantitatively synthesize the per-eye results of included studies using appropriate statistical methods. We calculated pooled estimates of treatment effects using random-effects or fixed-effects models, depending on the presence of heterogeneity. We performed sensitivity analyses to explore sources of heterogeneity and assess the robustness of the results. All analyses were conducted using statistical STATA software, with a significance level set at *p* < 0.05. Grading of Recommendations, Assessment, Development, and Evaluations (GRADE) assessment was applied using GRADEpro GDT [17].

## 3. Results

### 3.1. Literature Search

We searched databases and identified a total of one hundred and seventy-six citations. We screened titles and abstracts of 100 citations after removing the duplicates. 80 ineligible studies were excluded, leaving potentially 20 eligible for their full-text screening. All the 20 articles were retrieved, and 13 articles of them were excluded. Finally, 7 studies met the eligibility criteria for inclusion in this systematic review encompassing 606 eyes [18–24]. The PRISMA flow diagram is shown in Figure 1.

### 3.2. Characteristics of the Included Studies

A total of 7 studies comprising 606 eyes were included. Two of the seven included studies were conducted in Saudi Arabia, and another two were conducted in Turkey. Other locations of the included studies were Japan, India, and China. While the age values of the treatment groups were close to each other, the follow-up durations differed across groups. The characteristics of the included studies and baseline summary are shown in Tables 1 and 2.

### 3.3. Risk of Bias

One of the seven included studies was a randomized controlled trial, whose quality was assessed using the ROBINS 2 tool and was of moderate risk bias due to high risk in selective reporting of the outcome data and unclear information about the blinding of the outcome assessors and allocation concealment. The rest of the included studies were observation studies whose quality was assessed using the NOS tool. Three of the six included observational studies were of good quality, while the other three were of fair quality as their study groups were not comparable in the confounding factors (Supporting Tables 1S and 2S).

### 3.4. BCVA

**Figure 2:**
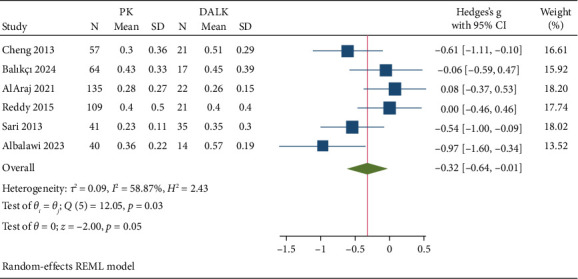
Best corrected visual acuity forest plot.

Six studies encompassing a total of 576 patients assessed BCVA. The pooled random-effect size showed that PK was inferior to DALK in terms of BCVA (Hedge's g: −0.32 with 95% CI [−0.64, −0.01], *p*=0.05) (Figure 2). The pooled data were severely heterogeneous (*I*^2^ = 58.87%; *p*=0.03). The leave-one-out sensitivity analysis for BCVA showed that Hedge's g ranged from −0.41 (95% CI: −0.75, −0.08) after excluding AlAraj et al. 2021 to −0.22 (95% CI: −0.51, 0.06) after excluding Albalawi et al. 2023 (Figure 1S). Galbraith plot to test for heterogeneity shows the asymmetrical distribution of studies around the pooled estimate, suggesting heterogeneity without any study outside 95% CI of the regression (Figure 2S).

### 3.5. BCVA at 6 Months

**Figure 3:**
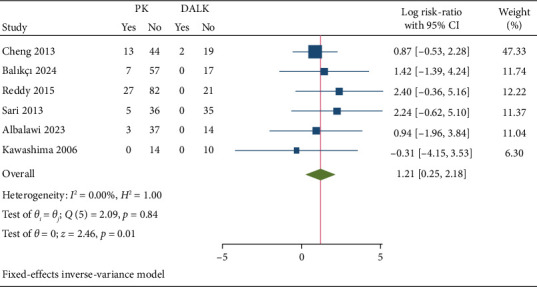
Graft rejection forest plot.

Two studies with a total of 128 patients assessed BCVA at 6 months. The pooled fixed-effect size showed that PK was inferior to DALK in terms of BCVA at 6 months (Hedge's g: −0.5 with 95% CI [−0.91, −0.1], *p*=0.02) (Figure 3S). The pooled data were homogeneous (*I*^2^ = 0%; *p*=0.77).

### 3.6. BCVA of 20\40 or Better

Three studies encompassing a total of 368 patients assessed BCVA of 20\40 or better. The pooled fixed-effect size showed that PK was inferior to DALK in terms of BCVA of 20\40 or better (log RR: −0.15 with 95% CI [−0.28, −0.01], *p*=0.03) (Figure 4S). The pooled data were homogeneous (*I*^2^ = 0%; *p*=0.81).

### 3.7. Endothelial Cell Density (Cells/mm^2^) at 1 year

Two studies encompassing 145 patients assessed endothelial cell density (cells/mm^2^) at 1 year. The pooled fixed-effect size showed that there was no difference between PK and DALK in terms of endothelial cell density at 1 year (Hedges's g: −0.09 with 95% CI [−0.43, 0.25], *p*=0.61) (Figure 5S). The pooled data were homogeneous (*I*^2^ = 52.3%; *p*=0.15).

### 3.8. Endothelial Cell Density (Cells/mm^2^) at 2 Years

Two studies with a total of 133 patients assessed endothelial cell density (cells/mm^2^) at 2 years. The pooled fixed-effect size showed that PK was associated with lower endothelial cell density rather than DALK at 2 years (Hedges's g: −0.79 with 95% CI [−1.16, −0.42], *p* < 0.001) (Figure 6S). The pooled data were homogeneous (*I*^2^ = 0%; *p* > 0.999).

### 3.9. Graft Rejection

Six studies encompassing a total of 443 patients assessed the risk of graft rejection. The pooled fixed-effect size showed that PK was associated with a higher risk of graft rejection compared to DALK (log RR: 1.21 with 95% CI [0.25, 2.18], *p*=0.01) (Figure 3). The pooled data were homogeneous (*I*^2^ = 0%; *p*=0.84).

### 3.10. Graft Failure

Two studies assessed the risk of graft failure in 105 patients. The pooled fixed-effect size showed no difference between PK and DALK in terms of risk of graft failure (log RR: −0.32 with 95% CI [−1.65, 1.01], *p*=0.64) (Figure 7S). The pooled data were homogeneous (*I*^2^ = 0%; *p*=0.45).

### 3.11. Recurrence of MCD

Five studies encompassing a total of 446 patients assessed the risk of recurrence. The pooled fixed-effect size showed that PK was higher than DALK in reducing the risk of recurrence (log RR: −0.88 with 95% CI [−1.45, −0.3], *p* < 0.001) (Figure 8S). The pooled data were homogeneous (*I*^2^ = 0%; *p*=0.8).

### 3.12. Corneal Infection

Two studies encompassing 238 patients assessed the risk of corneal infection. The pooled fixed-effect size showed no difference between PK and DALK in terms of risk of corneal infection (log RR: 0.58 with 95% CI [−1.09, 2.24], *p*=0.5) (Figure 9S). The pooled data were homogeneous (*I*^2^ = 0%; *p*=0.86).

### 3.13. Wound Dehiscence

Three studies with a total of 363 patients assessed the risk of wound dehiscence. The pooled fixed-effect size showed no difference between PK and DALK in terms of risk of wound dehiscence (log RR: 0.2 with 95% CI [−1.58, 1.98], *p*=0.83) (Figure 10S). The pooled data were homogeneous (*I*^2^ = 0%; *p*=0.82).

### 3.14. Glaucoma

Three studies encompassing a total of 368 patients assessed the risk of glaucoma. The pooled fixed-effect size showed no difference between PK and DALK in terms of risk of glaucoma (log RR: −0.02 with 95% CI [−1.00, 0.95], *p*=0.96) (Figure 12S). The pooled data were homogeneous (*I*^2^ = 0%; *p*=0.89).

### 3.15. Cataract

Four studies assessed the risk of cataract in 446 patients. The pooled fixed-effect size showed no difference between PK and DALK in terms of risk of cataract (log RR: 0.09 with 95% CI [−0.53, 0.71], *p*=0.78) (Figure 13S). The pooled data were homogeneous (*I*^2^ = 0%; *p*=0.84).

### 3.16. GRADE Assessment

Of the 13 assessed outcomes, nine were classified as critical outcomes, while the other four were important. The certainty of evidence was low in only two outcomes: (1) the endothelial cell density at 2 years and (2) the BCVA at 6 months. Certainty was downgraded two degrees in both of them due to the inclusion of observational studies in the evidence synthesis. Other outcomes' certainty was downgraded to three degrees due to one reason that downgraded all outcomes to two degrees: the inclusion of observational studies in evidence synthesis, while the other reason differed among the outcomes. Endothelial cell density (cells/mm^2^) at 1 year and BCVA were downgraded for the high heterogeneity (*I*^2^ > 40%) in the meta-analyses models. Graft rejection, perforation, and recurrence were downgraded for the serious imprecision of the pooled estimates (a small number of events < 300). The rest of the outcomes were downgraded for very serious imprecision, due to both the small number of events and the very wide 95% CI that includes beneficial/harmful effects (Table 3S).

## 4. Discussion

The transplantation method chosen for MCD is influenced by the severity of the patient's condition. DALK and PK have both achieved successful outcomes historically, but there are concerns surrounding lamellar surgery [25, 26]. DALK surgery has the benefit of maintaining the integrity of the eye, which may result in fewer complications before and after the operation. However, because macular dystrophy can impact the deeper layers of the cornea, it is believed that the visual prognosis after DALK could be worse than that following PK, and some research supports this idea [27]. Nonetheless, if the deep stroma and Descemet membrane are unaffected by the disease, DALK could be an appropriate choice. Techniques such as the Anwar big bubble method for Descemet's membrane have shown in some studies to yield visual outcomes that are comparable to or even superior to those of PK [28, 29].

In this study, we have compared PK to DALK in MCD patients. We found that DALK achieved better visual outcomes, encompassing high BCVA estimates and higher rates of achieving BCVA of 20/40 or better. However, graft rejection rates were significantly higher in the PK group compared to the DALK group; however, it was associated with low recurrence rates. Endothelial cell density differed at one year and two years, as there was no significant difference between the two groups at one year. In contrast, after two years, PK was associated with lower endothelial cell density. Graft failure rates were comparable between the two groups. Additionally, there was no statistically significant difference between the two groups in the incidence of postoperative complications: glaucoma, cataract, wound dehiscence, and corneal infections.

PK achieved lower BCVA estimates compared to the DALK technique. This opposes the findings by Chen et al. [30]. They found that PK achieved better BCVA and uncorrected visual acuity than the DALK group. Also, they did not find a statistically significant difference in the proportion of patients who achieved a BCVA of 20/40 or better. The difference in these metrics may be related to the difference in the indications for keratoplasty. Additionally, they have only included randomized controlled trials regardless of the indication. Although we included only MCD patients, there was a significant heterogeneity in the analysis. This heterogeneity could be explained by the inclusion of observational studies with only one randomized controlled trial, the difference in surgical experience, and the techniques utilized in the DALK. Therefore, we utilized SMD estimates rather than the weighted mean difference, such as the Chen et al. study. Our sensitivity analysis raises concerns about the robustness of our findings; however, when removed (Cheng 2013, Sari 2013, and Albalawi 2023), the results become insignificant, while others suggest the robustness of our findings. Further trials are needed to resolve this conflict.

Graft rejection rates were lower in the DALK group than in the PK group, which aligns with previous meta-analyses [30, 31]. Additionally, the recurrence rates were lower in the PK group compared to the DALK group. The recurrence of MCD in grafts that undergo keratoplasty has been documented in the literature [32–34]. These occurrences were attributed to donor keratocytes being replaced by genetically defective host cells [34]. In a previously discussed large retrospective series on PKP for MCD, the recurrence incidence was linked to the length of follow-up and inversely related to the size of the donor graft and recipient trephination [35]. Akova et al. noted recurrence times varying from 20 months to 30 years [32].

MCD involves the Descemet membrane and endothelium [36, 37]. After two years of follow-up, PK was associated with lower endothelial cell density, while at one year, there was no significant difference between the two groups. However, the evidence for this outcome is limited, as only two studies reported it. This could be due to the retrospective nature of the included studies and the inability to assess this outcome. Further prospective studies should investigate this outcome.

This is the first meta-analysis to compare PK to DALK in the treatment for MCD. However, it has some limitations: (1) a limited number of included trials, (2) some important outcomes are not reported, (3) the difference in follow-up, (4) the retrospective nature of the included studies, and (5) the difference in surgical experiences, DALK techniques among the included studies. Additional randomized controlled trials should include larger sample sizes and conduct assessments at more time points to improve outcome evaluation and address the under-reported outcomes.

## 5. Conclusion

Our findings indicate that DALK achieved superior visual outcomes, with higher rejection rates and lower recurrence rates compared to PK. No significant differences were observed between the two groups in terms of postoperative complications. Further randomized controlled trials with a larger sample size and longer follow-up should compare these two procedures.

## Figures and Tables

**Table 1:** Summary of the included studies.

Study ID	AlAraj 2021	Balıkçı 2024	Cheng 2013	Kawashima 2006	Reddy 2015	Sari 2013	Albalawi 2023
Year	2021	2024	2013	2006	2015	2013	2023

Study design	Retrospective, interventional case series	Retrospective cohort	Retrospective comparative case series	Age-matched control study	Retrospective comparative case series	Prospective randomized clinical trial	Retrospective cross-sectional, comparative study

Location of study	Saudi Arabia	Turkey	China	Japan	India	Turkey	Saudi Arabia

Study duration/duration of data collection	17 years	31 years	19 years	20 years	8 years	4 years	6.5 months

Target population	Patients with macular corneal dystrophy with a minimum of 12 months follow-up	Patients with macular corneal dystrophy with a minimum of 12 months follow-up	Patients with macular corneal dystrophy with a minimum of 12 months follow-up	Patients with macular corneal dystrophy (MCD) or lattice corneal dystrophy (LCD)	Patients with macular corneal dystrophy with a minimum of 12 months follow-up	Macular corneal dystrophy (MCD) without endothelial involvement	Patients with macular corneal dystrophy (MCD)

Intervention	Deep anterior lamellar keratoplasty (DALK)	Deep anterior lamellar keratoplasty (DALK)	Deep anterior lamellar keratoplasty (DALK)	Deep anterior lamellar keratoplasty (DALK)	Deep anterior lamellar keratoplasty (DALK)	Deep anterior lamellar keratoplasty (DALK)	Lamellar keratoplasty (LK)

DALK technique	Big bubble, Visco dissection, and hydro dissection	Big bubble technique	Layer-by-layer manual dissection	Lamellar surgical knives	Big bubble technique (in cases of BB failure baring of DM was achieved using a crescent blade and Vannas scissors)	Big bubble technique (in cases of BB failure baring of DM was achieved using layer-by-layer manual dissection)	NR

Control	Penetrating keratoplasty (PK)	Penetrating keratoplasty (PK)	Penetrating keratoplasty (PK)	Penetrating keratoplasty (PK)	Penetrating keratoplasty (PK)	Penetrating keratoplasty (PK)	Penetrating keratoplasty (PK)

Main findings	DALK had comparable medium-term visual and survival outcomes to PKP. DALK has the advantage of lower open sky intraoperative complications and lower graft rejection episodes	Both PK and DALK are equally effective in treating macular corneal dystrophy with similar visual, topographic, and survival outcomes. Although interface opacity occurs more frequently after DALK	PK more often improves the visual acuity of patients; however, it has many complications. DALK is associated with lower visual acuity outcomes but with fewer complications and more durable stability of the ocular surface compared to PK	Deep lamellar keratoplasty seems to be a safe alternative to PK for MCD and LCD	Visual and refractive outcomes are comparable between DALK and PK groups. DALK was superior to PK in its safety against postoperative complications such as endothelial rejection and secondary glaucoma.	Deep anterior lamellar keratoplasty (DALK) provided comparable visual and optical results as PK with less endothelial damage and eliminating endothelial rejection in MCD	PK operations resulted in better postoperative visual acuity compared to the LK group. The graft survival rate was slightly better for the PK group. PK was associated with a higher incidence of complications than the LK group.

*Sample size*							
Total	157 eyes of 100 patients	81 eyes of 59 patients	78 eyes of 51 patients	24 eyes	130 eyes of 104 patients	82 eyes of 54 patients	54 eyes of 46 patients
DALK	22 eyes (18 patients)	17 eyes	21 eyes	10 eyes	21 eyes of 20 patients	41 eyes	14 eyes of 14 patients
PK	135 eyes (82 patients)	64 eyes	57 eyes	14 eyes	109 eyes of 84 patients	41 eyes	40 eyes of 35 patients

**Table 2:** Baseline characteristics of the included studies.

Study ID	Study arms	Number of eyes	Age/years mean (SD)	Gender/male *n* (%)	Follow-up period in years mean (SD)	BCVA log MAR mean (SD)	UCVA log MAR mean (SD)	Donor corneal size/diameter in mm mean (SD)	Recipient corneal size/diameter in mm mean (SD)	Endothelial cell density/cells/m^2^ mean (SD)	Right eye: left eye: both eyes
AlAraj 2021	DALK	22	28.2 (7.5)	6 (27.3)	7.2 (6.2)	0.78 (0.25)	0.88 (0.2)	7.8 (0.28)	7.5 (0.35)	NA	9:5:4
	PK	135	31.5 (8.4)	39 (28.9)	9.7 (4.1)	0.75 (0.29)	0.82 (0.3)	7.7 (0.26)	7.3 (0.31)	NA	11:18:53

Balıkçı 2024	DALK	17	32.18 (7.92)	6 (35.3)	5.47 (3.35)	1.115 (0.43)	NA	NA	NA	NA	NA
	PK	64	39.98 (10.28)	27 (42.2)	7.85 (4.02)	1.40 (0.49)	NA	NA	NA	NA	NA

Cheng 2013	DALK	21	22.3 (10.9)	24 (47.1)	3.4 (2.1)	1.22 (0.59)	NA	NA	NA	NA	NA
	PK	57	37.9 (13.5)		5.7 (4.5)	1.49 (0.83)	NA	NA	NA	NA	NA

Kawashima 2006	DALK	LCD: 31 MCD: 10	MCD: 59 (15) LCD: 42 (10)	MCD: 6 (60) LCD: 23 (74.2)		MCD: 6 (60) LCD: 23 (74.2)	NA	NA	NA	LCD: 2446 (619) MCD: 2433 (85)	NA
	PK	LCD: 19 MCD: 14	MCD: 59 (12) LCD: 46 (11)	MCD: 9 (64.3) LCD: 19 (65.5)		MCD: −1.069 LCD: −0.811	NA	NA	NA	LCD: 2414 (384) MCD: N/C	NA

Reddy 2015	DALK	21	30 (11.9)	14 (70)	1.42 (0.73)	1.1 (0.6)	NA	7.6 (0.2)	8 (0.3)	NA	9:10:1
	PK	109	34 (11.5)	48 (57)	3.58 (2)	1.3 (0.6)	NA	7.6 (0.2)	8.1 (0.2)	NA	34:25:25

Sari 2013	DALK	41	29.7 (11.3)	9 (42.85)	2.54 (0.73)	1.30 (0.46)	1.34 (0.44)	NA	NA	2881 (449)	NA
	PK	41	33.0 (13.0)	16 (55.2)	2.6 (0.815)	1.36 (0.48)	1.40 (0.46)	NA	NA	2734 (549)	NA

Albalawi 2023	DALK	14	30.6 (5.9)	11 (78.6)	2.1 (0.8)	1.19 (0.11)	NA	NA	NA	NA	NA
	PK	40	32.4 (6.4)	29 (72.5)	1.9 (0.8)	1.1 (0.6)	NA	NA	NA	NA	NA

## Data Availability

Data sharing is not applicable to this article as no datasets were generated or analyzed during the current study.

## Supporting Information

Supporting Appendix: The search strategy.

Supporting Table 1S: ROB2 for RCTs.

Supporting Table 2S: NOS for cohort studies.

Figure 1S: Leave-one-out forest plot of best corrected visual acuity (BCVA).

Figure 2S: Galbraith plot of best corrected visual acuity (BCVA).

Figure 3S: Forest plot of best corrected visual acuity (BCVA) at 6 months.

Figure 4S: Forest plot of best corrected visual acuity (BCVA) of 20/40 or better.

Figure 5S: Forest plot of endothelial cell density (cells/mm^2^) at 1 year.

Figure 6S: Forest plot of endothelial cell density (cells/mm^2^) at 2 years.

Figure 7S: Forest plot of graft failure.

Figure 8S: Forest plot of recurrence of corneal macular dystrophy.

Figure 9S: Forest plot of corneal infection.

Figure 10S: Forest plot of wound dehiscence.

Figure 11S: Forest plot of microperforation.

Figure 12S: Forest plot of glaucoma.

Figure 13S: Forest plot of cataract.
