# Supplementary material for: Efficacy and Safety of Penetrating Keratoplasty and Deep Anterior Lamellar Keratoplasty in Corneal Macular Dystrophy: A Systematic Review and Meta-Analysis
Source: J Ophthalmol. 2025 Aug 6;2025:8867750. doi: 10.1155/joph/8867750 (PMC12349984; doi:10.1155/joph/8867750)
Supplement: Supporting Information — Additional supporting information can be found online in the Supporting Information section. [file 8867750.f1.docx]

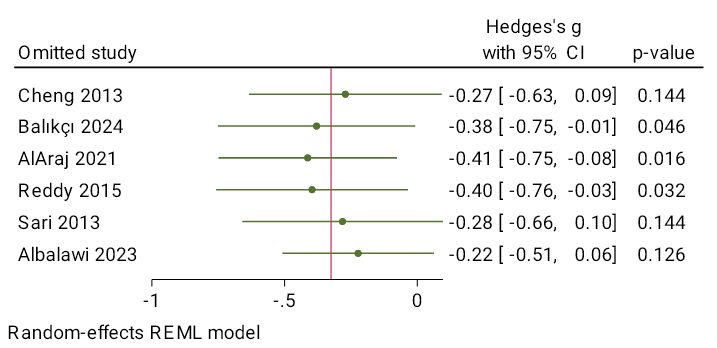


Figure. 1S Leave one out Forest plot of best corrected visual acuity (BCVA).


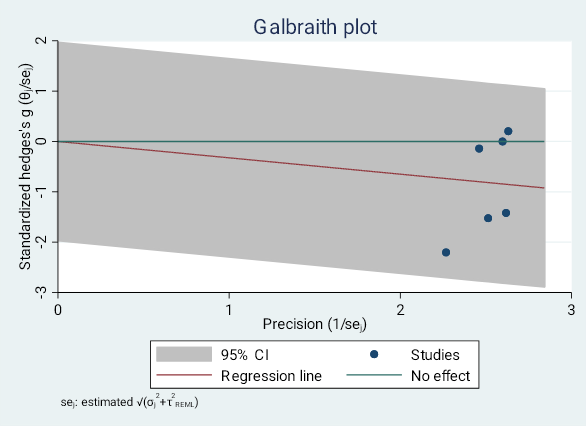


Figure. 2S Galbraith plot of best corrected visual acuity (BCVA).


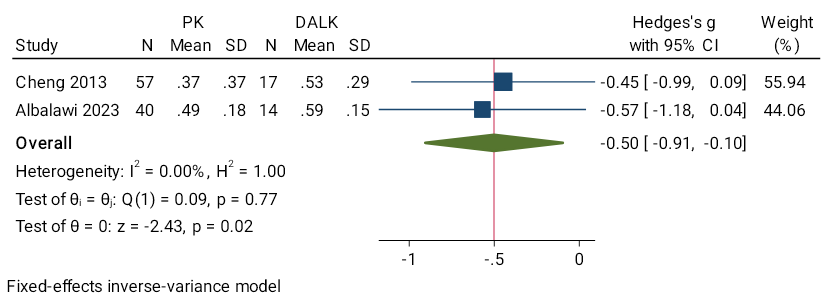


Figure. 3S Forest plot of best corrected visual acuity (BCVA) at 6 meters.


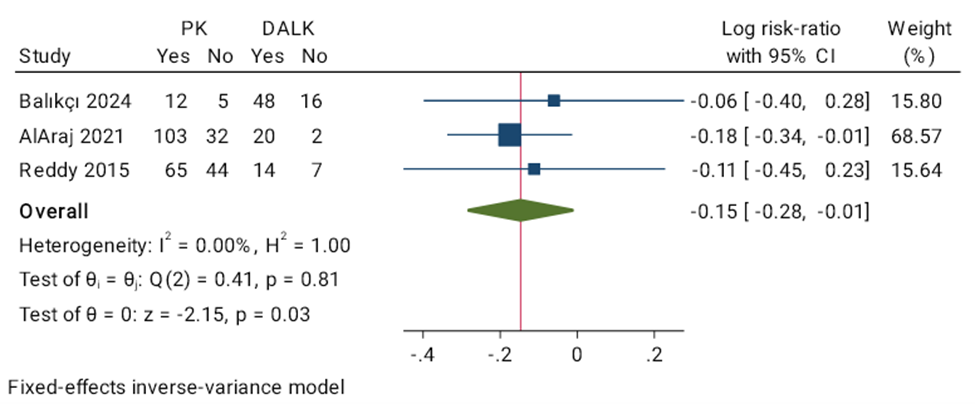


Figure. 4S Forest plot of best corrected visual acuity (BCVA) of 20/40 or better.


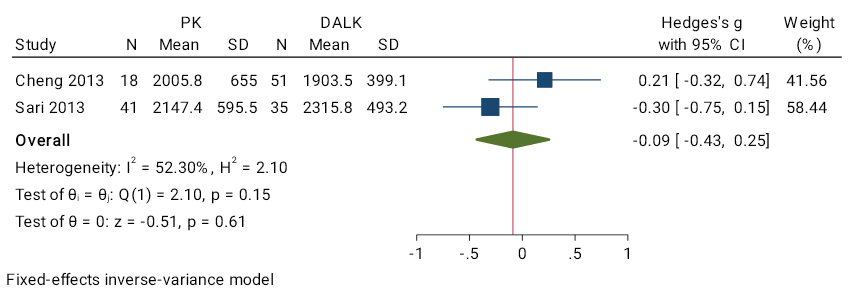
 Figure. 5S Forest plot of endothelial cell density (cells/mm2) at 1 year.

#
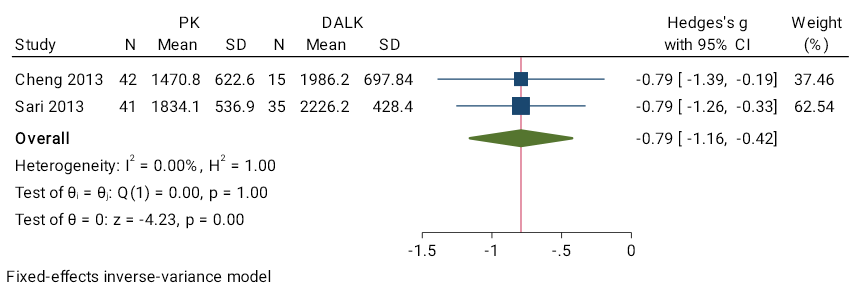


Figure. 6S Forest plot of endothelial cell density (cells/mm2) at 2 year.


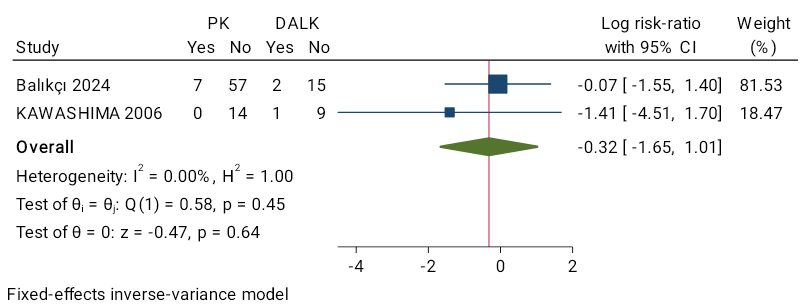


Figure. 7S Forest plot of graft failure.

#
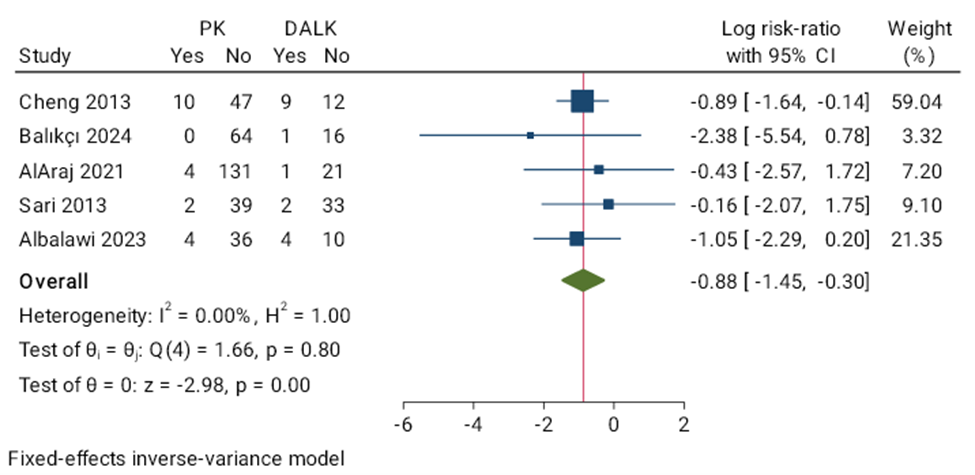


Figure. 8S Forest plot of recurrence of corneal macular dystrophy.


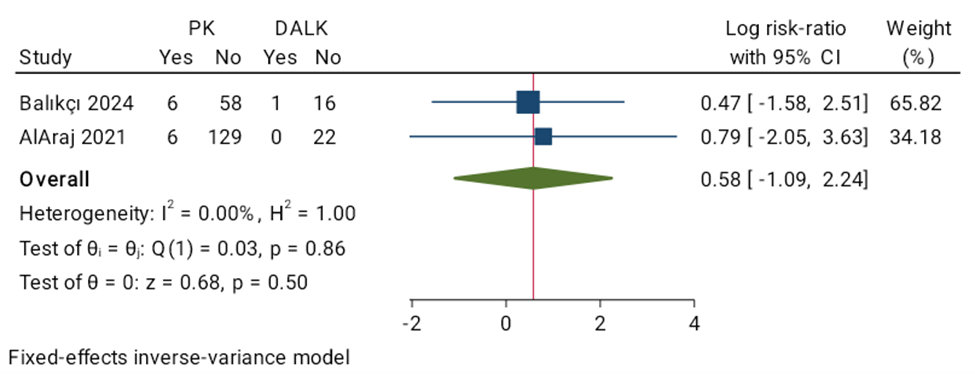


Figure. 9S Forest plot of corneal infection.


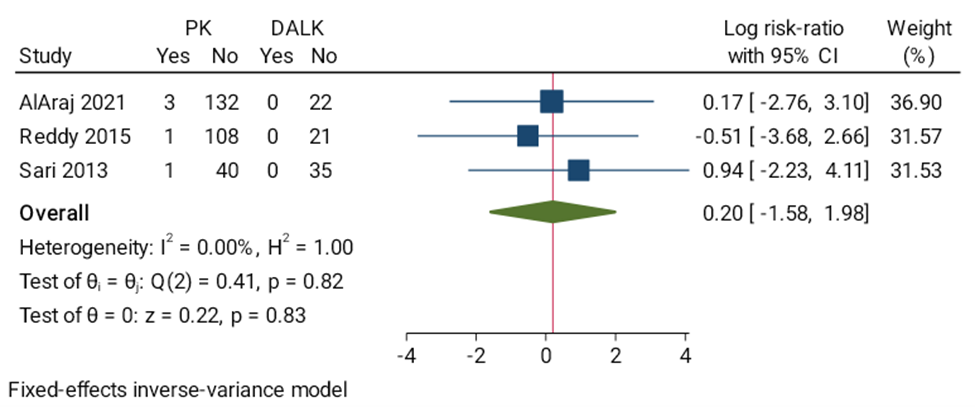


Figure. 10S Forest plot of wound dehiscence.


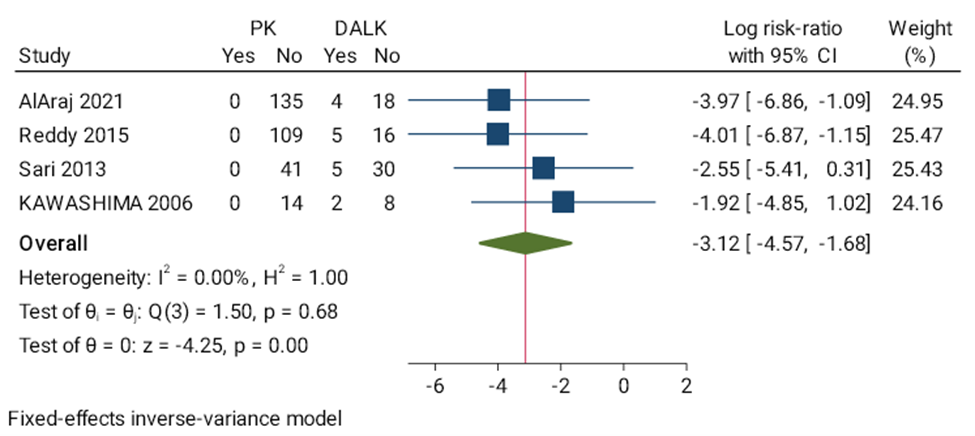


Figure. 11S Forest plot of micro-perforation.


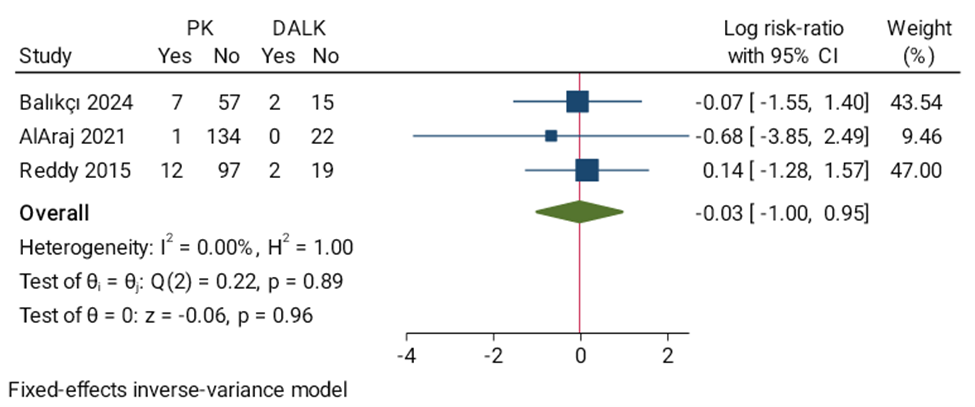


Figure. 12S Forest plot of glaucoma.


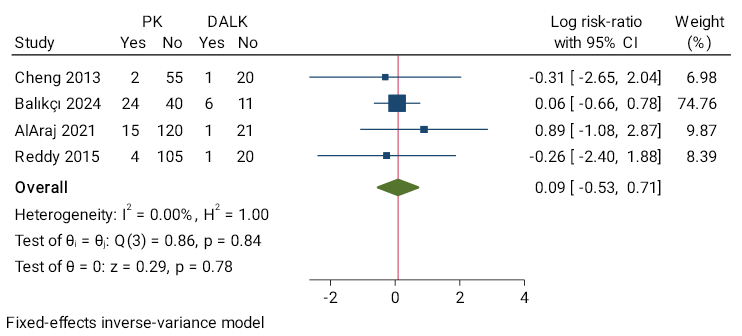


Figure. 13S Forest plot of cataract.

**Risk of bias assessment**

**Table 1S.** ROB2 for RCTs.

| Study ID | ROB: Sequence generation | | ROB: Allocation concealment | | ROB: Blinding of the study personnel and patients | | ROB: Blinding of outcome assessor | | ROB: Incomplete outcome data | | ROB: Selective outcome reporting | | ROB: other source of Bias | | Judgment Overall Risk of Bias |
| --- | --- | --- | --- | --- | --- | --- | --- | --- | --- | --- | --- | --- | --- | --- | --- |
|  | Judgment | Reason | Judgment | Reason | Judgment | Reason | Judgment | Reason | Judgment | Reason | Judgment | Reason | Judgment | Reason |  |
| Sari [6]2013 | low risk |  | unclear |  | low risk |  | unclear |  | high risk |  | low risk |  | low risk |  | moderate |

**Table 2S.** NOS for cohort studies.

| **Quality Assessment for Cohort Studies (NOS)** | | | | | | | | | | |
| --- | --- | --- | --- | --- | --- | --- | --- | --- | --- | --- |
| **Study ID** | **Selection** | | | | **Comparability** | | **Exposure** | | | **Score** |
|  | Representativeness of  the exposed cohort | Selection of the  nonexposed cohort | Ascertainment  of exposure | Demonstration that the outcome of  interest was not present at the start of the study | Study controls for age | Study controls for  any additional factor | Assessment of  outcome | Was follow-up long enough  for outcomes to occur | Adequacy of follow up  of cohorts | Quality Score |
| AlAraj 2021[1] | * | * | * | * | * | * |  | * | * | 8 |
| Balıkçı 2024[2] |  | * | * | * |  |  |  | * | * | 5 |
| Cheng 2013[3] |  | * | * | * | * |  |  |  | * | 5 |
| Kawashima 2006[4] |  | * | * | * | * | * |  |  | * | 6 |
| Reddy 2015[5] |  | * | * | * | * |  |  |  | * | 5 |
| Yara 2023[7] |  | * | * | * | * |  |  | * | * | 6 |

Table 3S. GRADE assessment

**Question:** PK compared to DALK for Corneal macular dystrophy

| **Certainty assessment** | | | | | | | **№ of patients** | | **Effect** | | **Certainty** | **Importance** |
| --- | --- | --- | --- | --- | --- | --- | --- | --- | --- | --- | --- | --- |
| **№ of studies** | **Study design** | **Risk of bias** | **Inconsistency** | **Indirectness** | **Imprecision** | **Other considerations** | **PK** | **DALK** | **Relative (95% CI)** | **Absolute (95% CI)** |  |  |
| **Best corrected visual acuity** | | | | | | | | | | | | |
| 6 | non-randomised studies | not serious | serious^a^ | not serious | not serious | none | 446 | 130 | - | SMD **0.32 SD lower** (0.64 lower to 0.01 lower) | ⨁◯◯◯ Very low | CRITICAL |
| **endothelial cell density (cells/mm2) at 1y** | | | | | | | | | | | | |
| 2 | non-randomised studies | not serious | serious^a^ | not serious | not serious | none | 59 | 86 | - | SMD **0.09 SD lower** (0.43 lower to 0.25 higher) | ⨁◯◯◯ Very low | CRITICAL |
| **endothelial cell density (cells/mm2) at 2y** | | | | | | | | | | | | |
| 2 | non-randomised studies | not serious | not serious | not serious | not serious | none | 83 | 50 | - | SMD **0.79 SD lower** (1.16 lower to 0.42 lower) | ⨁⨁◯◯ Low | CRITICAL |
| **BCVA at 6m** | | | | | | | | | | | | |
| 2 | non-randomised studies | not serious | not serious | not serious | not serious | none | 97 | 31 | - | SMD **0.5 SD lower** (0.91 lower to 0.1 lower) | ⨁⨁◯◯ Low | CRITICAL |
| **graft rejection** | | | | | | | | | | | | |
| 6 | non-randomised studies | not serious | not serious | not serious | serious^b^ | none | 55/325 (16.9%) | 2/118 (1.7%) | **RR 3.35** (1.28 to 8.75) | **40 more per 1,000** (from 5 more to 131 more) | ⨁◯◯◯ Very low | CRITICAL |
| **cataract** | | | | | | | | | | | | |
| 4 | non-randomised studies | not serious | not serious | not serious | very serious^b,c^ | none | 45/365 (12.3%) | 9/81 (11.1%) | **RR 1.09** (0.58 to 2.03) | **10 more per 1,000** (from 47 fewer to 114 more) | ⨁◯◯◯ Very low | CRITICAL |
| **Glaucoma** | | | | | | | | | | | | |
| 3 | non-randomised studies | not serious | not serious | not serious | very serious^b,c^ | none | 20/308 (6.5%) | 4/60 (6.7%) | **RR 0.97** (0.36 to 2.65) | **2 fewer per 1,000** (from 43 fewer to 110 more) | ⨁◯◯◯ Very low | CRITICAL |
| **micro-perforation** | | | | | | | | | | | | |
| 4 | non-randomised studies | not serious | not serious | not serious | serious^b^ | none | 0/299 (0.0%) | 16/88 (18.2%) | **RR 0.04** (0.01 to 0.18) | **175 fewer per 1,000** (from 180 fewer to 149 fewer) | ⨁◯◯◯ Very low | CRITICAL |
| **wound dehiscence** | | | | | | | | | | | | |
| 3 | non-randomised studies | not serious | not serious | not serious | very serious^b,c^ | none | 4/284 (1.4%) | 0/78 (0.0%) | **RR 1.22** (0.20 to 7.24) | **0 fewer per 1,000** (from 0 fewer to 0 fewer) | ⨁◯◯◯ Very low | CRITICAL |
| **Recurrence** | | | | | | | | | | | | |
| 5 | non-randomised studies | not serious | not serious | not serious | serious^b^ | none | 20/337 (5.9%) | 17/109 (15.6%) | **RR 0.41** (0.23 to 0.73) | **92 fewer per 1,000** (from 120 fewer to 42 fewer) | ⨁◯◯◯ Very low | IMPORTANT |
| **graft failure** | | | | | | | | | | | | |
| 2 | non-randomised studies | not serious | not serious | not serious | very serious^b,c^ | none | 7/78 (9.0%) | 3/27 (11.1%) | **RR 0.72** (0.19 to 2.74) | **31 fewer per 1,000** (from 90 fewer to 193 more) | ⨁◯◯◯ Very low | IMPORTANT |
| **corneal infection/ keratitis** | | | | | | | | | | | | |
| 2 | non-randomised studies | not serious | not serious | not serious | very serious^b,c^ | none | 12/199 (6.0%) | 1/39 (2.6%) | **RR 1.78** (0.33 to 9.48) | **20 more per 1,000** (from 17 fewer to 217 more) | ⨁◯◯◯ Very low | IMPORTANT |
| **BCVA of 20/40 or better** | | | | | | | | | | | | |
| 3 | non-randomised studies | not serious | not serious | not serious | very serious^b,c^ | none | 180/261 (69.0%) | 82/107 (76.6%) | **RR 0.86** (0.75 to 0.98) | **107 fewer per 1,000** (from 192 fewer to 15 fewer) | ⨁◯◯◯ Very low | IMPORTANT |

**CI:** confidence interval; **RR:** risk ratio; **SMD:** standardised mean difference

#### Explanations

a. I-squared < 40%

b. Few number of events (< 300)

c. The confidence interval includes beneficial/harmful effect

**Search strategy**

**PubMed** = 66 ( All fields)

compar* AND (lamell* AND penet*) AND (“Hereditary Corneal Dystrophy” OR “Hereditary Corneal Dystrophies” OR “Corneal Dystrophies” OR “Corneal Dystrophy” OR “Groenouw Dystrophies” OR “Groenouw's Dystrophies” OR “Groenouw Dystrophies” OR “Groenouws Dystrophies” OR “Corneal Stromal Dystrophy” OR “Corneal Stromal Dystrophies” OR “Corneal Granular Dystrophy” OR “Corneal Granular Dystrophies” OR “Corneal Macular Dystrophy” OR “Corneal Macular Dystrophies”)

**Scopus** = 57 (Title-Abstract-Keywords)

TITLE-ABS-KEY ( compar* AND ( lamell* AND penet* ) AND ( "Hereditary Corneal Dystrophy" OR "Hereditary Corneal Dystrophies" OR "Corneal Dystrophies" OR "Corneal Dystrophy" OR "Groenouw Dystrophies" OR "Groenouw's Dystrophies" OR "Groenouw Dystrophies" OR "Groenouws Dystrophies" OR "Corneal Stromal Dystrophy" OR "Corneal Stromal Dystrophies" OR "Corneal Granular Dystrophy" OR "Corneal Granular Dystrophies" OR "Corneal Macular Dystrophy" OR "Corneal Macular Dystrophies" ) )

**WOS** = 51 (All fields)

ALL=(compar* AND (lamell* AND penet*) AND (“Hereditary Corneal Dystrophy” OR “Hereditary Corneal Dystrophies” OR “Corneal Dystrophies” OR “Corneal Dystrophy” OR “Groenouw Dystrophies” OR “Groenouw's Dystrophies” OR “Groenouw Dystrophies” OR “Groenouws Dystrophies” OR “Corneal Stromal Dystrophy” OR “Corneal Stromal Dystrophies” OR “Corneal Granular Dystrophy” OR “Corneal Granular Dystrophies” OR “Corneal Macular Dystrophy” OR “Corneal Macular Dystrophies”))

**Cochrane** = 2 ( All fields)

compar* AND (lamell* AND penet*) AND (“Hereditary Corneal Dystrophy” OR “Hereditary Corneal Dystrophies” OR “Corneal Dystrophies” OR “Corneal Dystrophy” OR “Groenouw Dystrophies” OR “Groenouw's Dystrophies” OR “Groenouw Dystrophies” OR “Groenouws Dystrophies” OR “Corneal Stromal Dystrophy” OR “Corneal Stromal Dystrophies” OR “Corneal Granular Dystrophy” OR “Corneal Granular Dystrophies” OR “Corneal Macular Dystrophy” OR “Corneal Macular Dystrophies”)
